# Late metachronous cerebral metastasis of pancreatic adenocarcinoma of the tail of the pancreas: a case report

**DOI:** 10.1186/s13256-022-03314-w

**Published:** 2022-04-04

**Authors:** Kyriakos Papadimitriou, Daniel Kiss-Bodolay, Abderrahmane Hedjoudje, Diego San Millan, Alexandre Simonin, Jean-Yves Fournier, Karen Huscher

**Affiliations:** 1 Department of Neurosurgery, Hospital of Sion, Av. Grand-Champsec 80, 1951 Sion, Switzerland; 2 Department of Clinical Neurosciences, Service of Neurosurgery Lausanne University Hospital, University of Lausanne, Rue du Bugnon 46, 1011 Lausanne, Switzerland; 3 Department of Radiology, Hospital of Sion, Avenue du Grand-Champsec 80, 1950 Sion, Switzerland

**Keywords:** Pancreatic cancer, Pancreatic adenocarcinoma, Cerebral metastasis

## Abstract

**Background:**

Pancreatic cancer is one of the leading causes of cancer mortality and one of the most lethal malignant neoplasms worldwide. It is known for its local tumor extension to the liver; other common sites include the lung, distant lymph nodes, and bone. Brain metastases are extremely rare and represent less than 0.6% of all brain metastases.

**Case report:**

We report the case of a 66-year-old Caucasian female known to have adenocarcinoma of the tail of the pancreas treated with chemotherapy. During follow-up, thoracoabdominal computed tomography scans did not reveal any residual tumor or any metastasis. Moreover, tumor markers were within normal limits. She presented to the emergency department of our institution following an episode of a generalized tonic–clonic seizure 5 years following the initial diagnosis. Brain magnetic resonance imaging revealed an expansive left frontal intraaxial lesion compatible with high-grade glioma. The patient underwent surgical treatment. Histological examination revealed pancreatic metastasis.

**Conclusions:**

Thought to be rare, metachronous cerebral pancreatic metastasis should be kept in mind in patients with pancreatic cancer. Early diagnosis and complete surgical resection play a key role in the survival of these patients.

## Introduction

Pancreatic cancer is one of the leading causes of cancer mortality and one of the most lethal malignant neoplasms worldwide [4]. Pancreatic cancer accounts for 4% of all deaths and is the seventh leading cause of cancer deaths in both sexes [4]. The estimated 5-year survival rate for pancreatic cancer remains dismal. Recently published data showed that, in recent decades, mortality has been increasing in the USA, Europe, and Asia [4, 10]. The mainstay of treatment remains radical resection combined with adjuvant radiotherapy and chemotherapy [28].

Pancreatic adenocarcinoma is known for its local tumor extension to the liver; other common sites include the lung, distant lymph nodes, and bone [23, 28]. Brain metastases are extremely rare and represent less than 0.6% of all brain metastases [5, 10].

We describe herein a case of late metachronous brain metastasis in a patient known for biologically and radiologically silent adenocarcinoma of the tail of the pancreas mimicking a high-grade primary brain tumor.

## Clinical presentation

**Fig. 1:**
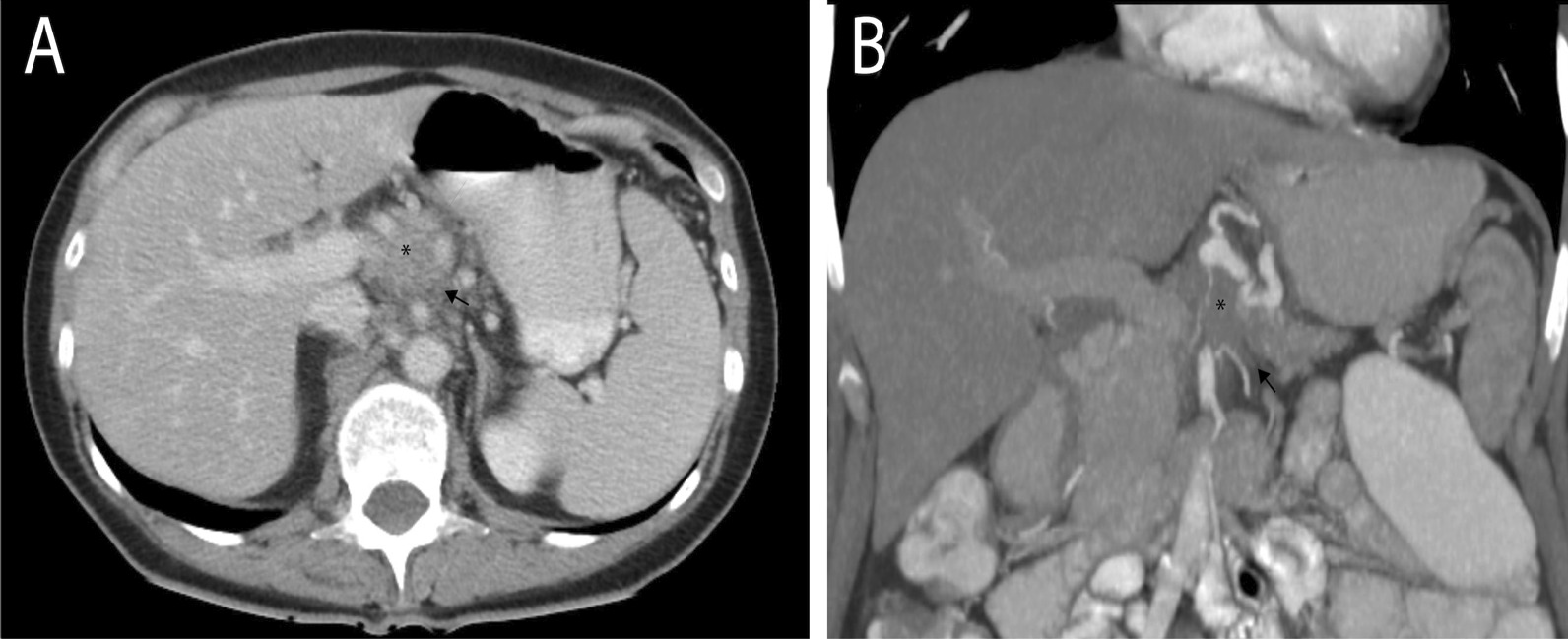
**A** Axial reconstruction of computed tomography (CT) scan showing the tumor of the pancreas (*) with infiltration of peripancreatic fat tissues (black arrow) **B** Coronal reconstruction

This 66-year-old right-handed Caucasian female was diagnosed 5 years ago (Fig. 1) with unresectable adenocarcinoma of the tail of the pancreas (T4N1M1). At the time of diagnosis, the tumor’s size was approximately 30 mm and it was encircling the celiac trunk, in contact with the splenic artery. It was associated with precaval and retrocaval lymphadenopathy. She therefore underwent needle biopsy, which revealed pancreatic adenocarcinoma. The carcinoembryonic antigen (CEA) and carbohydrate antigen (CA)19-9 were 21.3 μg/L and 900 kU/L respectively.

**Fig. 2:**
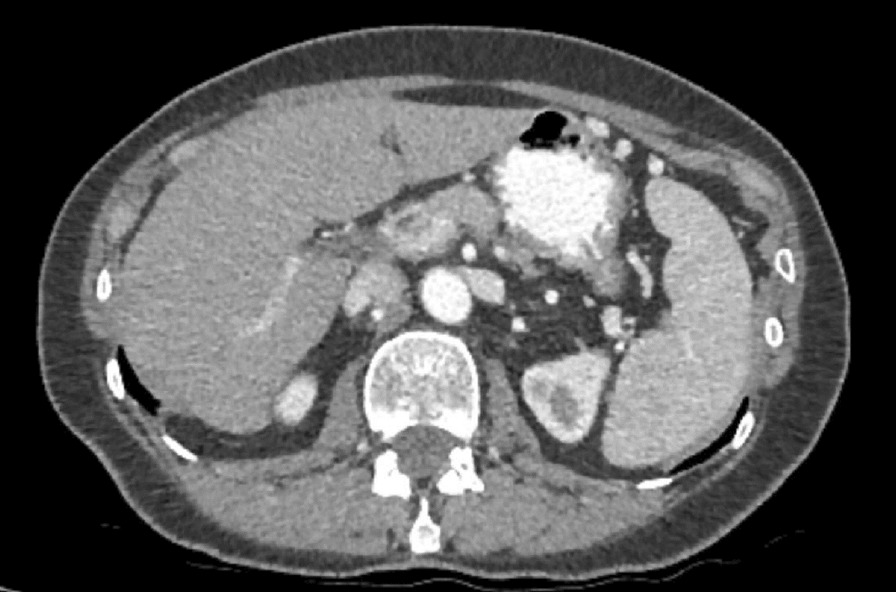
Posttreatment CT showing no residual pancreatic tumor

She was initially treated with two cycles of FOLFIRINOX and 5-fluorouracil. Due to intolerance to this regimen, the treatment was switched to a combination of Abraxane and gemcitabine with a good oncologic response. She received a total of ten cycles. During the last oncologic evaluation, the CEA and CA19-9 were within normal limits. Of note, adjuvant radiotherapy was not given to the patient. On regular follow-ups 4 years later, thoracoabdominal computed tomography (CT) revealed no detectable tumor or any metastatic lesions (Fig. 2).

**Fig. 3:**
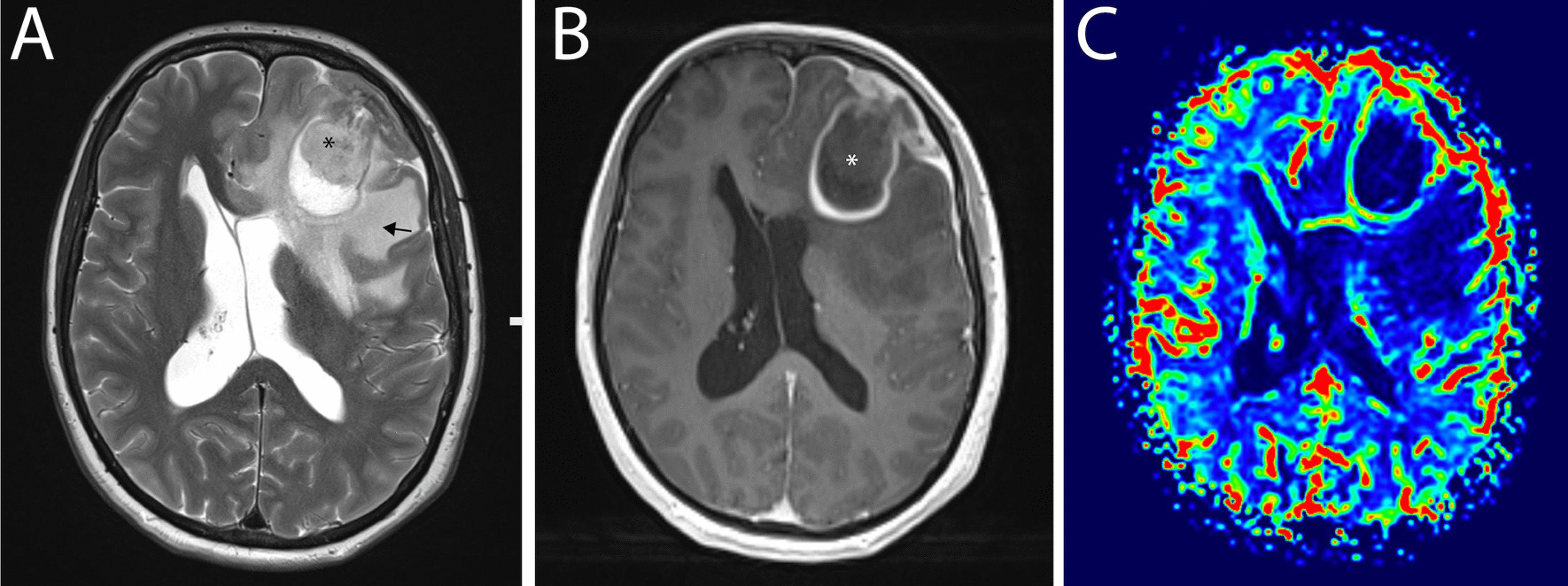
**A** Axial T2-w images show a cystic frontal lobe mass (asterisk) with evidence of significant surrounding vasogenic edema (black arrow). Brain compression and midline shift are evident on MRI. **B** Axial post-contrast T1-w images show the contrast-enhancing mass lesion (asterisk) throughout the left frontal with fairly well-encapsulated appearances. **C** Contrast-enhanced MRI perfusion imaging indicates high perfusion (green–red) in areas of viable tumor compared with baseline brain perfusion (black-blue)

Six months after the last oncologic follow-up, she presented to our institution with an episode of generalized tonic–clonic seizure, which was treated with levetiracetam. Other than headaches, the clinical examination did not reveal any neurological deficit. Neurological and physical examinations were unremarkable. Cerebral magnetic resonance imaging (MRI) showed a 5-cm cortical and white matter mass in the left middle frontal gyrus (Fig. 3). The mass had an extraaxial component attached to the dura matter adjacent to the frontal pole. The tumor had several large areas of central necrosis. Following gadolinium administration, avid contrast uptake was noted on the periphery of the lesion. T2-weighted images showed important vasogenic edema with mass effect on the frontal horn of the lateral ventricle that was provoking midline shift and subfalcine herniation. Laboratory analysis including liver and pancreatic function tests did not show any significant abnormality. The full blood count was unremarkable. Due to the good response to treatment, the rarity of cerebral metastasis in pancreatic cancer patients, the normal value of the last CA19-9, and these radiological findings, the most likely diagnosis was high-grade glioma.

The patient underwent left frontal craniotomy and surgical removal of the tumor. There was a dense, solid part as shown on the figures that was adhered on the dura without any areas of necrosis or any macroscopic evidence of thrombosed vessels.

**Fig. 4:**
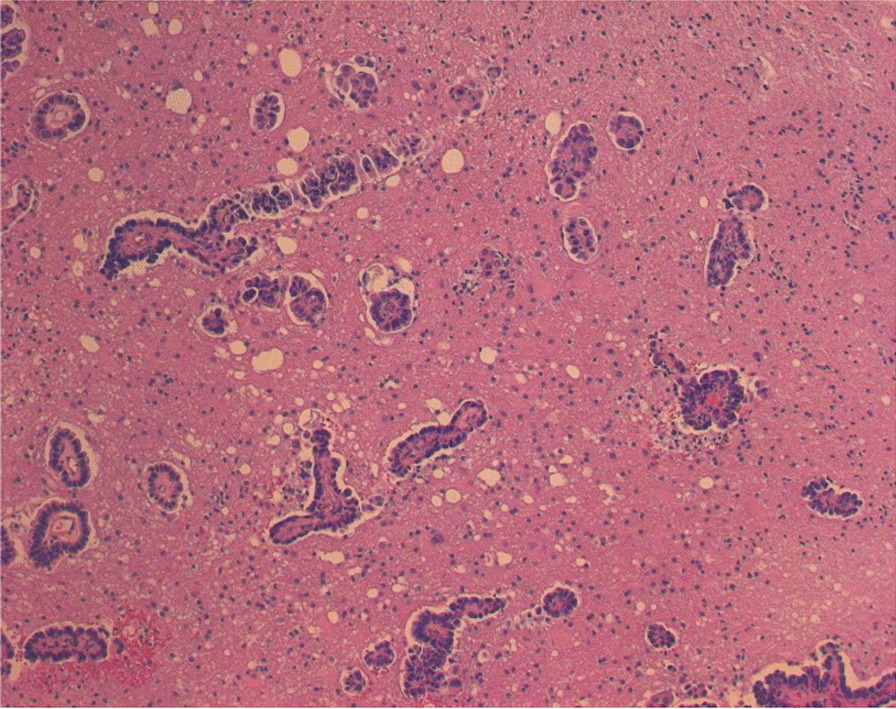
Microscope analysis revealed the tumor to be a moderately differentiated adenocarcinoma surrounded by extracellular matrix (H and E)

The postoperative course was uneventful. She remained stable with no neurological signs or symptoms. Histopathological analysis revealed cubic to cylindrical cells with focal marked atypia (Fig. 4). Immunohistochemistry revealed tumor cells diffusely positive for cytokeratin 7 and CEA. More than 50% of the cells were positive for TTF1, CDX2, and GATA3. There was no expression of cytokeratin 20, PAX8, WT1, or the estrogen and progesterone receptors. These results were compatible with a metastatic lesion of pancreatic adenocarcinoma.

**Fig. 5:**
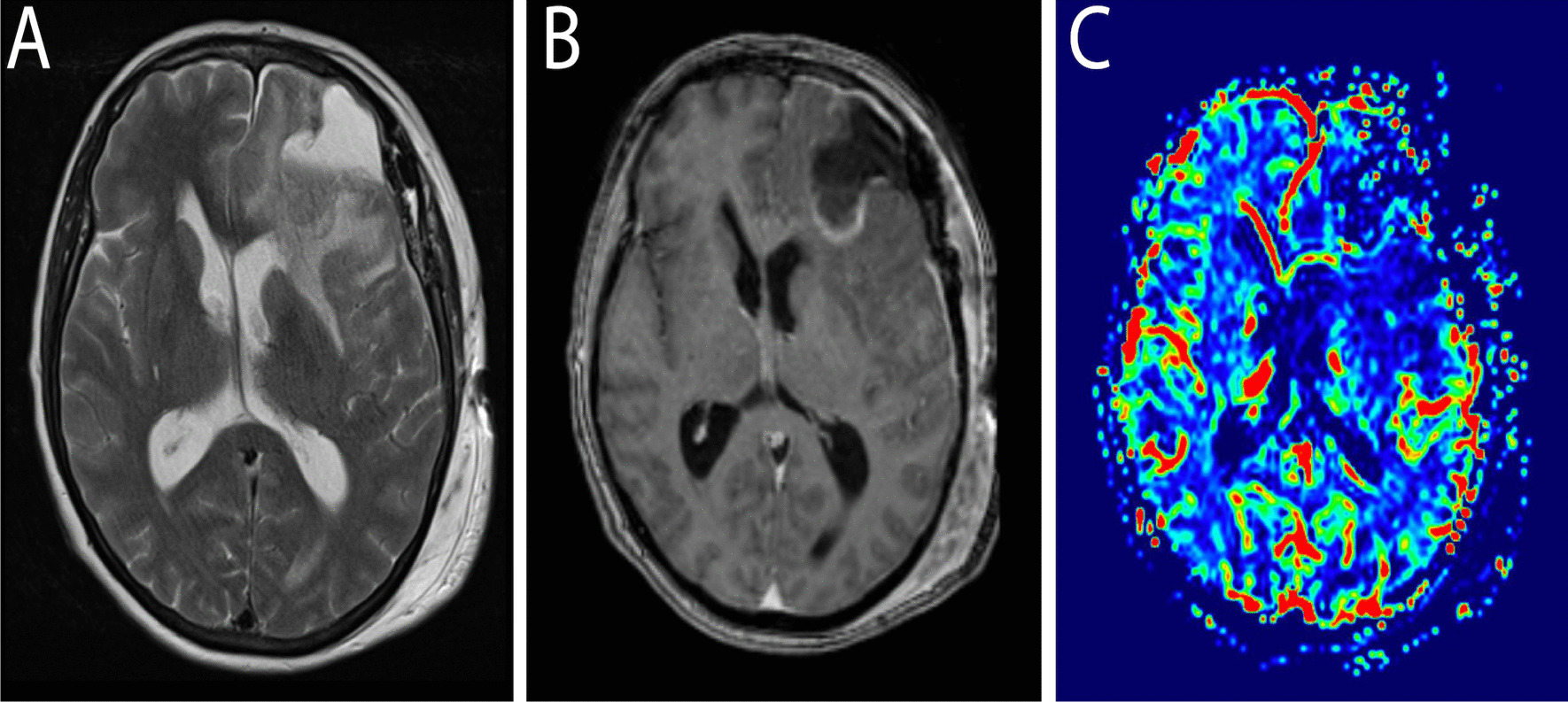
**A** Axial T2-w images show a reduction of the mass effect and persistence of surrounding vasogenic edema. **B** Axial postcontrast T1-w images show a reduction of the mass lesion. **C** Contrast-enhanced MRI perfusion imaging shows a reduction of high perfusion (green–red) areas

Immediate postoperative brain MRI revealed no complications (Fig. 5).

Notably, during hospitalization, a repeat CA19-9 was abnormally high (221 kU/l). A repeat thoracoabdominal CT and positron emission tomography (PET)-CT revealed bilateral pulmonary micronodules without any fluorodeoxyglucose (FDG) uptake. It did not, however, reveal any recurrent pancreatic tumor or metastatic disease. She was discharged home on postoperative day 5 on Keppra. She thereafter received a total of 30 Gy of adjuvant radiotherapy on the surgical bed. At 6-month follow-up, she was doing well without any radiological signs of tumor recurrence.

## Discussion

Pancreatic cancer, mostly adenocarcinoma, represents around 3% of all cancers diagnosed each year, affecting more frequently males older than 65 years [10, 23]. Due to its silent nature and late presentation of symptoms, the prognosis of pancreatic cancer remains poor with a 5-year survival rate of less than 4% [5, 23] and a median survival of 3.9–7.4 months. Surgical resection is the best chance for cure, but only 15–20% of patients are candidates for pancreatectomy [26]. Approximately 65% of pancreatic cancers occur in the head of the pancreas, 15% occur in the body and tail, and the remaining 20% may involve the entire gland [2, 26]. It has been previously shown that patients with body or tail lesions had decreased median survival, increased frequency of metastatic disease, and were less likely to undergo surgery because head lesions cause symptoms earlier [2, 26]. In contrast to published works, our patient had an unusually long disease-free survival despite the unresectability of the pancreatic tumor.

Approximately 59% of patients with unresectable pancreatic adenocarcinoma will develop distant metastasis [20]. Forty percent of these patients will be diagnosed with metastasis in the peritoneum, 38% in the liver, and 10% in the lung [20]. Metastasis to the central nervous system (CNS) from pancreatic carcinoma is extremely rare [1, 3, 6, 9, 13, 16, 19, 25, 27]. In a series of 1229 patients with pancreatic cancer, Park *et al.* reported only four cases of cerebral (0.32%) and three spinal metastasis (0.25%) [19]. Similarly, according to the Surveillance, Epidemiology, and End Results (SEER) database (2010–2013) including 13,233 patients with stage IV pancreatic cancer, the incidence of brain metastasis was 0.6% [18]. Recently, Luu *et al.* reported a cohort of 2492 patients with pancreatic cancer undergoing surgical treatment where four patients (0.2%) suffered from cerebral metastasis [12]. This low incidence rate likely reflects the short survival time of these patients, where death occurs before developing symptomatic CNS metastasis. Moreover, patients with advanced pancreatic cancer are not systematically investigated for CNS metastasis [8, 19], likely resulting in underestimation of the incidence of asymptomatic brain metastasis. This hypothesis is supported by autopsy studies that revealed rates of pancreatic cerebral metastasis of up to 8% [9, 10].

The interval between initial diagnosis of pancreatic cancer and brain metastases varies between 2 months to more than 6 years [19]. In the work from Park *et al.*, two patients presented with symptomatic brain metastasis 4–5 years following the initial diagnosis [19]. These two patients had concomitant lung or liver lesions at the time of diagnosis of cerebral metastasis. Our case has some similarities in terms of late metachronous presentation of the brain metastasis, but our patient was radiologically and biologically cured. Rarely, brain metastases can be the first manifestation of pancreatic cancer [5, 19].

The treatment of choice for cerebral brain metastases depends on the estimated prognosis and aim of treatment and may include surgical resection, whole-brain radiotherapy, and stereotactic radiosurgery [8, 14, 24]. The treatment options have been discussed in detail elsewhere [14]. It is worth mentioning that, in general, if the primary tumor is not well controlled, surgical treatment for brain metastasis will not improve the survival rate [14]. Similarly, Park *et al.* reported that the cause of death was not due to the nervous system metastasis [19]. However, as reported by Lemke *et al.*, resection of distant cerebral metastases originating from pancreatic adenocarcinoma can result in a significant increase in survival in patients with well-controlled primary disease [9, 10]. Lastly, there is no clear evidence for the use of adjuvant chemotherapy for brain metastasis due to pancreatic cancer [15]. In our case, the patient received adjuvant radiation with satisfactory clinical outcome.

MRI findings in patients with pancreatic cerebral metastasis usually revealed a single intraaxial, cyst-like lesion, with an area of necrosis, irregular ring enhancement, and perifocal edema [14, 19]. Moreover, pancreatic metastasis can be present as an isolated, small epidural mass to diffuse leptomeningeal spread without solid mass [3]. Rarely, patients can present with multiple metastatic lesions with simultaneous leptomeningeal carcinomatosis [21]. Our case presented some unique radiological findings. In contrast to primary pancreatic cancer that is hypoperfused on perfusion-weighted MRI [29], in our patient the cerebral lesion was hyperperfused. Moreover, in the majority of cases reported, there was either an extra- or intraaxial component. However, in this case, there was mainly an intraaxial mass with dura invasion and a smaller extraaxial component. Therefore, our initial differential diagnostic was glioblastoma versus gliosarcoma. Moreover, pancreatic metastasis can mimic radiologically other pathologies such as cerebral amyloid angiopathy or hemangioblastoma [7, 17].

The incidence of brain metastases from pancreatic adenocarcinoma is currently expected to increase because of prolonged survival due to improved treatment protocols. All patients with suspicion of metastatic CNS lesions should have cerebral CT scan and MRI [10–14]. Neurologic symptoms led to brain imaging in all cases reported in literature, since routine brain MRI is not recommended for staging of pancreatic cancer [12]. However, to improve outcomes, early detection is crucial. Recently, it was suggested that screening MRI for brain metastases should be performed in asymptomatic patients with extremely elevated CA19-9 levels [12]. Therefore, the current trend to perform contrast-enhanced cerebral imaging is advised by some authors even in asymptomatic patients [22].

## Conclusion

We present a rare case of a late metachronous brain metastasis mimicking a high-grade glioma in a patient with a biologically and radiologically cured cancer of the tail of the pancreas. In our experience, surgical resection followed by adjuvant radiotherapy may be a good option in carefully selected patients. However, this remains an isolated case report, therefore further prospective studies need to be carried out. Lastly, clinicians should be aware of this rare entity, since its incidence may increase nowadays because of prolonged survival due to improved treatment regimens.

## Data Availability

Not applicable.
